# Application of Nanoemulsions (W/O) of Extract of *Opuntia oligacantha* C.F. Först and Orange Oil in Gelatine Films

**DOI:** 10.3390/molecules25153487

**Published:** 2020-07-31

**Authors:** Salvador Omar Espino-Manzano, Arely León-López, Gabriel Aguirre-Álvarez, Uriel González-Lemus, Laurette Prince, Rafael Germán Campos-Montiel

**Affiliations:** 1 Department of Food, Universidad Tecnológica de la Huasteca Hidalguense. Carr. Huejutla-Chalahuiyapa km. 3.5 S/N, C.P. Huejutla, Hidalgo 43000, Mexico; drsalvadorespino2020@gmail.com (S.O.E.-M.); lslprincess@gmail.com (L.P.); 2 Department of Food, Instituto de Ciencias Agropecuarias, Universidad Autónoma del Estado de Hidalgo. Av. Universidad Km. Rancho Universitario C.P. Tulancingo, Hidalgo 43600, Mexico; arlely@hotmail.com (A.L.-L.); aguirre@uaeh.edu.mx (G.A.-Á.); urielgonzalezlemus@gmail.com (U.G.-L.)

**Keywords:** packaging, gelatine film, radical inhibition, xoconostle, mechanical properties

## Abstract

Over the past decade, consumers have demanded natural, completely biodegradable active packaging serving as food containers. Bioactive plant compounds can be added to biopolymer-based films to improve their functionality, as they not only act as barriers against oxidation, microbiological, and physical damage, they also offer functionality to the food they contain. A water-in-oil (W/O) nanoemulsion was produced by applying ultrasound to xoconostle extract and orange oil, and was incorporated into gelatine films in different proportions 1:0 (control), 1:0.10, 1:0.25, 1:0.50, 1:0.75, and 1:1 (gelatine:nanoemulsion). The nanoemulsions had an average size of 118.80 ± 5.50 nm with a Z-potential of −69.9 ± 9.93 mV. The presence of bioactive compounds such as phenols, flavonoids, and betalains in the films was evaluated. The 1:1 treatment showed the highest presence of bioactive compounds, 41.31 ± 3.71 mg of gallic acid equivalent per 100 g (GAE)/100g for phenols, 28.03 ± 3.25 mg of quercetin equivalent per 100 g (EQ)/100g flavonoids and 0.014 mg/g betalains. Radical inhibition reached 72.13% for 2,20-azino-bis-3-ethylbenzothiazoline-6-sulphonic acid (ABTS), and 82.23% for 1,1-diphenyl-2-picrylhydrazyl (DPPH). The color of the films was influenced by the incorporation of nanoemulsions, showing that it was significantly different (*p* < 0.05) to the control. Mechanical properties, such as tensile strength, Young’s modulus, and percentage elongation, were affected by the incorporation of nanoemulsified bioactive compounds into gelatine films. The obtained films presented changes in strength and flexibility. These characteristics could be favorable as packaging material.

## 1. Introduction

During the past decade, food researchers focused their studies on replacing packaging materials derived from petrochemical-based plastics that dominate the market due to their low cost, functionality, and availability, with their extensive use becoming a waste management problem, since most of these plastics are single-use [1]. The new trends focused on the development of biopolymer films with high biodegradability and functional properties. Gelatine is a biopolymer used for its film-forming properties and good barrier functionality; it acts as a plasticizer with a main effect of lowering the glass transition temperature, i.e., the temperature at which the mechanical properties change due to the internal movements of the polymer chains, thereby obtaining more flexible films [2].

Gelatine is widely used in the pharmaceutical and food industries for the manufacture of a large number of products, mainly capsules, tablet coatings, and microcapsules [3]. Some examples of food applications are desserts [4,5], edible films [6,7,8,9], confectionery [10,11], clarifying agents [12], emulsifiers [13], and stabilizers [14]. To enhance the antioxidant and antimicrobial properties and extend the shelf-life of edible biopolymer-based films, natural antioxidants and antimicrobial agents are used as alternatives to synthetic preservatives (antimicrobials such as benzoates and sulfur dioxide delay the growth of bacteria, yeast and molds; antioxidants, such as butylated hydroxy toluene (BHT), butylated hydroxy anisole (BHA), and propyl gallate, control the breakdown of fats and oils; antienzymatic preservatives, such as erythorbic acids, block enzymatic processes) [15,16]. The bioactive compounds used in edible films include enzymes, bacteriocins, triclosan, plant-seed extracts, plant extracts, essential oils, α-tocopherol, organic acids. and chitosan [17,18]. In this work, gelatine films were mixed up with the entire xoconostle fruit extract.

Xoconostle is a Mexican fruit whose bioactive extracted compounds were studied over the last ten years, found to be a rich source of antioxidant compounds, such as phenolic acids and betalains; cactus pear (xoconostle) acid was also used as a natural preservative to help avoid food decomposition [19,20]. The use of bioactive compounds in the food industry is limited because they are frequently affected by factors such as pH, water activity, oxygen, enzyme activity, light, and temperature [21,22]. Bioactive compounds were found to conserve antioxidant and antimicrobial activities by an encapsulation process, such as the water/oil (W/O) nanoemulsion, which involves a transparent or translucent system with a particle size ranging from 10 to 100 nm; these compounds are thermodynamically unstable and are produced by high-energy methods, such as high shear agitation, high homogenization, and high ultrasound pressures. They were shown to improve bioavailability and avoid instability, especially when are exposed to diverse environmental conditions [23,24,25,26].

Another source of bioactive compounds is essential oils, e.g., orange oil, which has a marked antioxidant stability attributed to the presence of α-tocopherol, d-limonene, and some flavones [27,28]. Additionally, the encapsulation of active compounds in essential oils could lead to a longer maintenance of their usefulness by a controlled release of these compounds. Other studies on the antimicrobial activity of films enriched with essential oils identified bergamot [29], lemon, and sweet and bitter orange [30] as potential candidates. This study was carried out to compare antioxidant activities, color values, and mechanical properties of gelatine polymers as biodegradable films formed by combining nanoemulsions W/O of xoconostle extracts and orange oil. The purpose of this comparison was to detect the most functional concentration for the desired mechanical properties and antioxidant activities of the developed films.

## 2. Results and Discussion

### 2.1. Stability of the Nanoemulsions

The orange oil and xoconostle extract (W/O) nanoemulsions exhibited an average droplet size of 118.80 ± 5.50 nm. These results were consistent with [21], who found a drop diameter in the nanoemulsion (W/O) orange oil/xoconostle aqueous extract of 118 ± 5.50 nm, and [31] obtained a media droplet size of 94 ± 8 nm in same nanoemulsion (orange oil/xoconostle aqueous extract). Other authors [32] prepared an oil-in-water nanoemulsion of curcumin and oil, with an average droplet size of 141.6 ± 15.4 nm. The Z-potential was directly related to the droplet size of the nanoemulsion, indicating the stability of the system [33,34]. The obtained value for the orange oil and xoconostle extract (W/O) nanoemulsion was −69.9 ± 9.93 mV. Previous studies [35] showed that when preparing a water-in-oil (W/O) nanoemulsions with sodium alginate, lemon essential oil, and Tween 80 using sonication, a nanoemulsion with an acceptable stability was obtained with a Z-potential of −55.8 ± 6.4 mV. A potential of −85 mV was found in [21], where a water-in-oil (W/O) nanoemulsion was prepared, with a continuous phase of orange essential oil and a disperse phase of xoconostle cactus pear extract, using soy lecithin as the emulsifier; this showed excellent stability. Similar results were presented by [33], who found −106 ± 5 mV, indicating stability against phase separation. The negative Z-potential value for a nanoemulsion could be a result of the adsorption of hydroxyl ions at the oil–water interface and subsequent development of hydrogen bonds between these ions and the ethylene oxide groups of the emulsifier, with the resulting net charge contributing to the system’s stability [36,37,38].

### 2.2. Bioactive Compounds and Antioxidant Activity

The bioactive compounds were phenols (144.10 ± 0.96 mg GAE/100 g) and flavonoids (90.01 ± 0.76 mg QE/100 g) in the xoconostle extract. The amounts of phenol and flavonoids may depend on the variety of the fruit used [21]. The final concentration of phenols in the xoconostle/orange oil nanoemulsion (W/O) was 174.22 ± 0.21 mg GAE/100 g, and the final concentration of flavonoids was 105.01 ± 0.42 mg QE/100 g. The concentrations of bioactive compounds measured in the film samples are shown in Table 1. The 1:1 treatment showed the highest amounts of phenols (41.31 ± 3.71 mg GAE/100g) and flavonoids (28.03 ± 3.25 mg QE/100g), which were significantly different to the other treatments (*p* < 0.05); the authors of [39,40] found similar results in films made from fish jelly with green tea extract and zeina films with *Zataria multiflora Boiss* essential oil respectively.

The antioxidant activities of the films expressed as % of razino-bis (3-ethylbenzothiazoline-6-sulphonic acid) (APTS) inhibition and inhibition of the radical 1,1-diphenyl-2-picrylhydrazyl (DPPH) are shown in Table 1. The 1:1 treatment reached a percentage of inhibition of the ABTS radical of 72.13%. The authors of [41] achieved a maximum inhibition value for the same radical of 57% for gelatine-based films with turmeric extract. Antioxidant activity was measured as percent inhibition of the DPPH radical, showing a similar trend to inhibition of the ABTS radical. Inhibition of 82.23% was found in the 1:1 treatment, showing significant differences (*p* < 0.05) between the 1:1, 1:0.75, and 1:0.50 treatments compared to the 1:0.25 and 1:0.10 treatments. The authors of [39] made fish gelatine films by adding green tea extract, reaching a maximum of 65% inhibition for the DPPH radical, while [42] made films based on fish gelatine with longan seed extract, achieving an inhibition of 41%. The antioxidant activities (ABTS and DPPH) showed significant improvements (*p* < 0.05) with the addition of the nanoemulsions. The percentage of ABTS inhibition resulted in films loaded with nanoemulsions comparable to [43,44] for gelatin–chitosan-based films loaded with boldo or guarana extracts and nanoemulsified active compounds. The authors of [38] made gelatin–chitosan films loaded with a nanoemulsion of garlic oil/cinnamaldehyde and α-tocopherol, with values of inhibition of 0.22 ± 0.02 and 2.63 ± 0.12 mmol trolox equivlent/g film, respectively. The antioxidant activity of the films could potentially be attributed to the phenolic acids and terpenoids coming from the orange oil and xoconoxtle extract [21,45]. Xoconostle is rich in polyphenols, as reported by [22,46,47]. Synergistic effects could exist in addition to this, with contributions from the residual free amino groups of the gelatine molecule occurring by reacting with free radicals, forming stable macromolecular radicals and ammonium groups [38].

### 2.3. Quantification of Betalains

**Figure 1:**
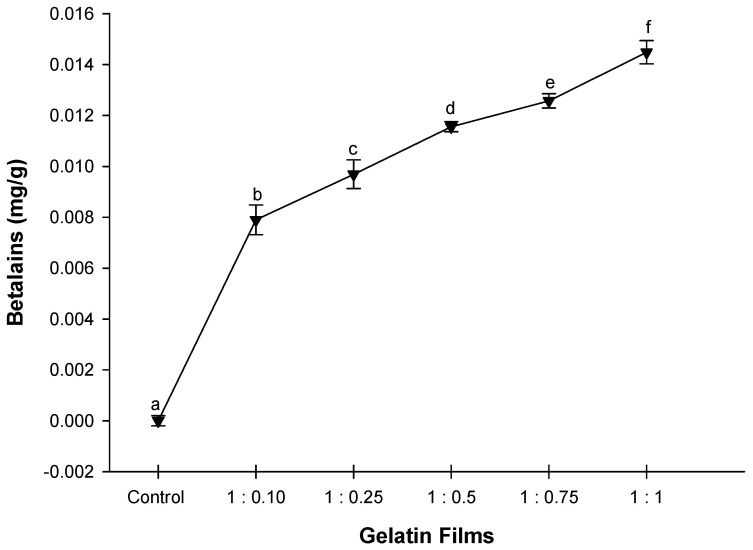
Concentration of betalains present in gelatine films with added nanoemulsion (W/O) xoconstle fruit extract–orange oil. Values with the same letter within the line are not statistically different values according to the Tukey test (*p* < 0.05).

Figure 1 shows the concentrations of betalains in the different treatments. The 1:1 treatment showed the highest concentration of betalains, with a value of 0.014 mg/g. Betalains are pigments that are responsible for the red or yellow color of fruits belonging to the order of *Caryophyllales*. One of these plants, xoconostle cactus pear (*Opuntia ficus-indica* L.), contains betalains in the fruits, particularly betacyanins in the purple variety and betaxanthins in the orange variety. Measurement of the total content of betalains in extracts from fruits such as xoconostle is important because it is an important source of pigments, which not only has coloring potential, but is also an excellent source of dietary antioxidants when used as a food ingredient [48].

### 2.4. Color in Films

The L* (brightness) and a* and b* (coordinates) color values obtained from the gelatine film samples loaded with the xoconostle–orange oil extract nanoemulsion (W/O) are shown in Table 2. Color parameters in the production of edible films are important because they define the acceptance of a product. Incorporation of the W/O nanoemulsion in the gelatine film had a significant effect on its final color. The L* parameter was different for the control and the samples containing the nanoemulsion. The a* parameter showed negative values, indicating that the films presented greenish shades.

**Figure 2:**
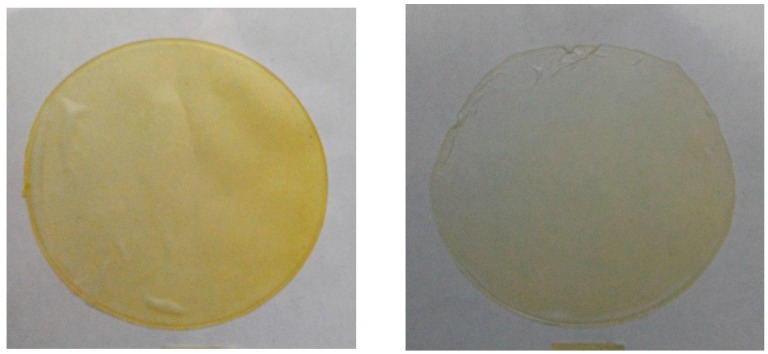
Colors of films with the addition of nanoemulsions 1:01 and 1:1.

The yellow hue produced by the films was evaluated with the parameter b*, giving positive values. This yellow hue decreased as the amount of nanoemulsion decreased, with the 1:0.1 sample showing the lowest yellow hue and presenting a b* value of 8.16 ± 0.40 in comparison with the 1:1 sample (Figure 2). These results were consistent with [49], who made fish gelatine films by incorporating citric essential oils and finding that the color of the film was affected by the addition of oils, resulting in less luminous films and yellowish tones.

### 2.5. Mechanical Properties

**Figure 3:**
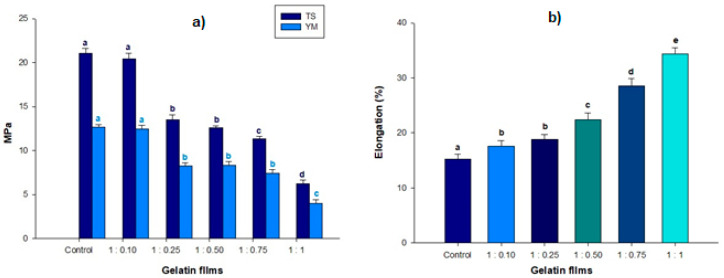
Mechanical properties of the different films with W/O nanoemulsions (control, 1:0.10, 1:0.25, 1:0.50, 1:0.75, and 1:1). (**a**) Dark blue bars indicate Young’s modulus (YM) in gelatine films and light blue bars indicate tensile strength in films where results are expressed in mega pascals (MPa). (**b**) The bars indicate the percentage of elongation in the gelatine films. ^a,b,c,d,e^ Different letters mean statistically different values according to the Tukey test (*p* < 0.05).

Mechanical properties of the films are affected by the interaction of the compounds used for formation with proteins that define the structure. These compounds could be water, hydrocolloids, and other possible additives, such as plasticizers and antioxidant agents [50]. Young’s modulus (E), tensile strength (TS), and elongation percentage (E%) values of gelatine films containing different volumes of nanoemulsion are shown in Figure 3.The control films displayed the highest (*p* < 0.05) tensile strength (TS), with the incorporation of nanoemulsions in the films causing a significant decrease in TS compared to the control (*p* < 0.05); these results were similar to those found by [40], with 19.0 MPa reported in other studies when they added lipophilic species, demonstrating a decrease in TS values of biopolymer-based films. At the same time, E% values in films loaded with nanoemulsions were significantly increased (*p* < 0.05) [51,52]. The 1:1 treatment showed the highest elongation (34.35%) with respect to the rest of the treatments. Research performed by [53,54] demonstrated that the incorporation of extrapolymeric materials strengthened the protein matrix, making the films more elastic. Figure 3a shows the values measured in the films for Young’s modulus [55]. It was observed that the stiffness of the films decreased as the concentrations of the nanoemulsified compounds increased, with the lowest value being 3.97 M for the 1:1 treatment. The Young’s modulus is a parameter that has a direct relationship with film stiffness. The Young’s modulus results were similar to those reported by [56], where a reduction in corn films occurred with the addition of safflower oil encapsulate.

### 2.6. Thermal Properties and Fourier Transform Infrared (FTIR)

The enthalpies of the 1:1 and 1:0.75 treatments were 303.6 and 302.7 J/g respectively, indicating that the addition of nanoemulsion may have caused an additional plasticizing effect alongside that given by glycerol. The higher the concentration of the nanoemulsion added to the gelatine film, the higher the energy needed to break its stability, since, by exerting plasticizing action, it did not allow protein chains to interact with each other. Authors such as those of [55,56,57,58,59] observed similar behavior when using protein as a surfactant to achieve the incorporation of oil in gelatine films. The addition of nanoemulsion to the gelatine films had a positive effect because there was less interaction between proteins, making the films more stable.

These results concurred with those obtained from FTIR; the films demonstrated better mechanical properties, making them more elastic and resistant. The spectra shown by the films (Figure 3a), both from the control and those containing the nanoemulsion at different concentrations, showed similar bands but with different intensities due to the presence of nanoemulsified compounds. Representative bands of gelatine were detected in the control film, as well as in the nanoemulsion films. Amide I was detected at a wavelength of 1641 cm^−1^, representing the stretching vibrations of the carbonyl groups (C = O) and the stretching and flexing vibrations of the NH group. The CN group stretching vibrations representative of amide II were detected at 1548 cm^−^^1^. Amide III was detected at 1248 cm^−1^, representing the stretching vibrations of the C–N group and the deformation of the NH group from amide bonds. Amide A was detected at a wavelength of 3295 cm^−1^, which detects NH group tension vibrations. The wavelength of 2946 cm^−1^ was representative of amide B, related to the asymmetric stretching of the CH_2_ group [60,61,62]. The spectra of the nanoemulsion films showed lower amplitudes of the amide A band (3170–3400 cm^−1^) due to homogeneous distribution of the nanoemulsion in the gelatine matrix, which was influenced by the presence of soy lecithin powder used as a surfactant when incorporating the nanoemulsion into the filmogenic solution. Similar results were found by [63], who used soy lecithin as a surfactant to incorporate essential oils into protein biofilms. The representative band of the OH groups was found at 1037 cm^−1^, which not only indicated the presence of the glycerol used as a plasticizer [64], but also the difference in intensity between each of the samples. This can be attributed to the presence of both phenolic compounds and betalains found in the xoconostle filtrate used as the aqueous phase of the nanoemulsion [19,20].

**Figure 4:**
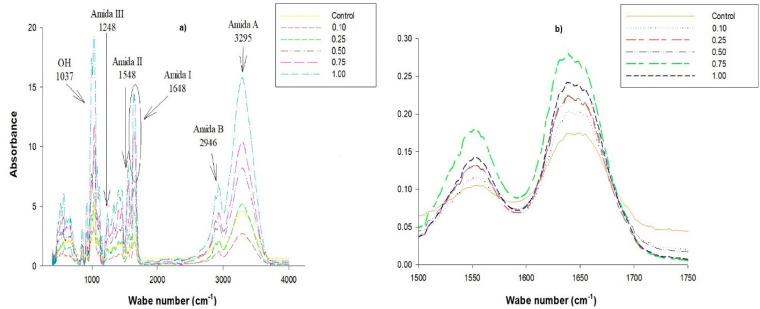
(**a**) FTIR spectra of gelatine films with the presence of bioactive nanoemulsified compounds. (**b**) FTIR spectra of gelatine films showing the representative band of amide II and C = C double bonds.

The bands shown at 1650–1670 cm^−1^ (Figure 4b) suggest the presence of double bonds between carbons; these bands are amplified due to the presence of the orange oil used to prepare the nanoemulsion, which contained terpenes [26]. The presence of the nanoemulsified bioactive compounds prevented the reorganization of the gelatine chains, which was consistent with the mechanical properties; when the concentration of the nanoemulsion increased, the gelatine films showed greater resistance, as they were much more flexible.

## 3. Materials and Methods

### 3.1. Fruits

**Figure 5:**
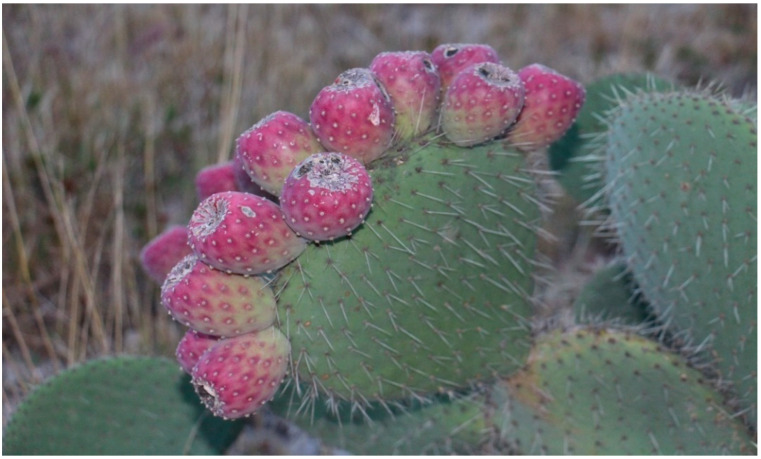
Xoconostle plant (*O. Oligacantha C.F. Först var “Ulapa”*).

Cactus pear fruits, “xoconostle” (*O. Oligacantha C.F. Först var “Ulapa”*), were harvested at Tezontepec de Aldama, Hidalgo, Mexico, when they reached physiological maturity, as shown in Figure 5.

### 3.2. Xoconostle Bioactive Compounds Extract

The ultrasound-assisted extraction of bioactive compounds from xoconostle was performed in an ultrasonic generator (130 W, 20 kHz, VCX130, VIBRACELL, Newtown, CT, USA). The conditions of the ultrasound-assisted extraction were 10 min of ultrasound, 40% methanol aqueous solution (PQ06121, Fermont, Monterrey, Mexico), and a solvent ratio of 1:20 (g/mL). The extracts obtained were filtered (Whatman paper No. 2, Sigma-Aldrich, St. Louis, MO, USA) and centrifuged at 17,500× *g* for 15 min at 20 °C (HERMLE Z 232 MKII, Labortechnik GmbH, Wehingen, Germany) the supernatant was the xoconostle extract, which was used for the experiment. This was refrigerated at 4 °C until further analysis [47]. The xoconostle pulp obtained showed similar physicochemical properties to those reported by [20] The total sugar content was 13.52 ± 0.07 mg glucose/mL pulp, soluble solids were 6.05 ± 0.30 g/100 g, the pH was 3.58 ± 0.7, moisture was 94.08% ± 0.02%, and ash was 0.82% ± 0.01%.

### 3.3. Preparation and Characterization of the Nanoemulsion

Nanoemulsions were prepared following the previously published method described in [65], with some modifications. The continuous phase (70%) was orange essential oil (W282510, Sigma-Aldrich, San Louis, MO, USA), the dispersed phase was xoconostle bioactive compound extract (20%), and soy lecithin (429415, Sigma-Aldrich, St. Louis, MO, USA) (10%) was the surfactant. Both phases were stirred using an ultrasonic processor (Ultrasonic Vibra-Cell VCX 130 Sonics, Newton, CT, USA). A probe 6 mm long was used and 20 intervals (59 s/interval) of sonication and recesses of 10 s were executed to obtain the necessary drop size. The ultrasonic processor was used at 80% amplitude at a frequency of 20 kHz, and the mixture was placed in an ice bath to avoid temperature increases during mixing. The nanoemulsion was refrigerated at 4 °C until further use, and was used within the first 12 h of preparation. Zetasizer was used (Nano-ZS2000 Model, Malvern Instruments Ltd., Malvern, Worcestershire, UK) to determine the Z-potential of the nanoemulsion by placing the sample in a cell coupled to gold electrodes (Dip Cell, Malvern Instruments Ltd., Worcestershire, UK). The droplet size distribution of the nanoemulsion was determined using the dynamic laser light scattering technique using Zetasizer equipment (Nano-ZS2000 Model, Malvern Instruments Ltd., Worcestershire, UK) by placing the sample in a glass cell. Five replicates were considered for each formulation.

### 3.4. Film Preparation

The film was made according to [39], with some modifications. A total of 0.76 g of gelatine powder (Merck, Darmstadt, Germany) was hydrated in 10.9 mL of cold water and heated at 60 °C for 10 min (Scorpion Scientific, Cd. México, Mexico). Simultaneously, 0.76 g of soy lecithin powder (429415, Sigma-Aldrich St. Louis, MO, USA) was hydrated in 9 mL of water and heated for 3 min at 70 °C, then 3.9 g of glycerol (104057, Merk, Edo. México, Mexico) was added, followed by addition of the lecithin/glycerol mixture to the melted gelatine. The mixture obtained was homogenized in an ULTRA-TURRAX (IKA, Shanghai, China) at 3200 rpm for 3 min. Subsequently, the nanoemulsion was added drop-by-drop in the following proportions of gelatine:nanoemulsion: 1:0, 1:0.10, 1:0.25, 1:0.50, 1:0.75, and 1:1.0. To form the films, 15 mL of the mixture was molded in a Petri dish (100 mm × 15 mm) at 25 °C for 24 h over P_2_O_5_.

### 3.5. Extraction of Active Compounds from the Films

Extraction was carried out according to a study by [66], with modifications. One gram of film was mixed with 10 mL of ethanol (100983, Merck, Edo. México, Mexico) and distilled water (1:1 *v*/*v*), and placed in an ultrasonic bath for 20 min at 45 °C. Next, this mixture was centrifuged at 17,500 rpm for 15 min at 20 °C in a Z 36 centrifuge (HERMLE Labortechnik GmbH, Wehingen, Germany). Then, the pellet obtained was mixed with 20 mL of aqueous solution of acetone (100012, Merck, Edo. México, Mexico) in a ratio of 70:30 *v*/*v* for 10 min and centrifuged at 10,000 rpm for 10 min at 4 °C. Both supernatants (ethanol and acetone extracts) were combined, stirred for 5 min, and centrifuged under the conditions described above. Samples were kept at 4 °C under dark conditions until analysis.

### 3.6. Total Phenols

The evaluation of total phenols was carried out using the methodology described by [67], with some modifications. Each sample was dissolved in distilled water (1:10), 0.5 mL of which was placed in test tubes protected from light, with addition of 2.5 mL of Folin-Ciocalteu reagent (F9252, Sigma-Aldrich, St. Louis, MO, USA), which was previously prepared (1:10) and mixed with 4 mL of 7.5% solution of sodium carbonate (Na_2_CO_3_) (PQ17881 Fermont, Monterrey, Mexico) for 7 min. The mixture was left for 120 min, and then it was read at a 760 nm wavelength in a spectrophotometer (JENWAY, Model 6705, Dunmow, UK). Distilled water was used as a blank. The experimental data were expressed in mg of gallic acid (G7384 Sigma-Aldrich, St. Louis, MO, USA) equivalents for 100 g of film (mg GAE/100 g film).

### 3.7. Total Flavonoids

The determination of total flavonoids was carried out by the method described by [68] with some modifications. A solution of aluminum trichloride (AlCl_3_) (449,598 Sigma-Aldrich St. Louis, MO, USA) in 2% methanol (PQ06121 Fermont, Monterrey, Mexico) was used, with 2 mL of the sample mixed with 2 mL of methanolic solution of AlCl_3_ added. The mixture was then left standing for 20 min in the dark, followed by the sample being read at a wavelength of 415 nm using a spectrophotometer (JENWAY, Model 6705. Dunmow, UK). The blank was methanol (PQ06121, Fermont, Monterrey, Mexico). The results were expressed in mg of quercetin (1592409, Sigma-Aldrich, St. Louis, MO, USA) equivalents for 100 g of film (mg QE/100 g film).

### 3.8. Total Betalains Determination

To quantify total betalains, the protocol established by [19] was followed, whereby 1 mL of sample was taken and mixed with 20 mL of 20% methanol (PQ06121, Fermont, Monterrey, Mexico). Absorbance readings were taken using a spectrophotometer (JENWAY, Model 6705, Dunmow, UK) at a wavelength of 483 nm (betaxanthines) and 538nm (betacyanins). The results were expressed in mg/g of film.

### 3.9. ABTS Assay

Antioxidant activity was measured using the 2,20-azino-bis (3-ethylbenzothiazoline-6-sulphonic acid) (A1888, Sigma-Aldrich, St. Louis, MO, USA) radical following [69] by reacting 10 mL of 7 mM ABTS solution with 10 mL of 2.45 mM (K_2_S_2_O_8_) potassium persulfate (216224, Sigma-Aldrich, St. Louis, MO, USA). The mixture was stirred for 16 h in a container in complete darkness. The absorbance was adjusted with 20% ethanol to obtain a value of 0.7 ± 0.1. A total of 200 μL of sample was added to 2 mL of ABTS solution and allowed to react for 6 min; absorbance was measured at 734 nm in a spectrophotometer (JENWAY, Model 6705, Dunmow, UK).

### 3.10. DPPH Assay

To determine antioxidant activity by inhibition of the DPPH radical, the DPPH solution was prepared by dissolving 7.8 mg of 1,1-diphenyl-2-picrylhydrazyl radical (D9132, Sigma-Aldrich, St. Louis, MO, USA) in 80% methanol (PQ06121, Fermont, Monterrey, Mexico). The solution was agitated for 2 h in the dark [70]. Then, 2.5 mL aliquots of 6.1 × 10-5M methanolic DPPH solution were added to glass tubes and reacted with 0.5 mL of sample. The tubes were left to stand in the dark for 30 min. Subsequently, the samples were measured at 515 nm using a spectrophotometer (JENWAY, Model 6705, Dunmow, UK). The blank was an 80% methanol (PQ06121, Fermont, Monterrey, Mexico) aqueous solution. The percent inhibition was calculated using Equation (1).

(1) $$\%\;\mathrm{Inhibition}=\frac{\mathrm{Initial}\;\mathrm{absorbance}-\mathrm{Final}\;\mathrm{absorbance}}{\mathrm{Initial}\;\mathrm{absorbance}}\times 100$$

### 3.11. Color Values of the Films

Color values were determined using a CM-508d colorimeter (Minolta, Tokyo, Japan) to evaluate the parameters L* (lightness), a* (green to red) and b* (blue to yellow). Five measurements were taken for each film, and 10 films were examined per treatment per day [31].

### 3.12. Mechanical Properties of Gelatine/Nanoemulsions Films

The measured mechanical properties were Young’s modulus, the elongation percentage, and the tensile strength according to the standard method, American Society for Testing Materials (ASTM) D882-95a [71]. The texture analyzer was used to load the cell with 50 kg (Brookfield CT3, Harlow, UK). The separation between the clamps was 0.5 mm and the equipment was operated at a speed of 0.10 mm/s. The films were cut in the form of a rectangle (10 cm long and 1 cm wide). The rectangles were kept at 57% relative humidity (NaBr).

### 3.13. Scanning Calorimetry (DSC) and FTIR Measurements

The differential scanning calorimetry technique (DSC) was used to measure the melting temperature (Tm) and denaturation enthalpy (ΔH) parameters, which were important in the evaluation of the stability of the biopolymer gelatine-based films. The methodology in [3] was followed with some modifications. A Q 2000 Q-series DS instrument kit equipped with a RCS90 refrigerated cooling system and TA 2000 universal analysis software was used. The equipment was calibrated using indium as the standard metal. The samples of the films were weighed (1 ± 0.1 mg) in aluminum trays and hermetically sealed. The trays with the samples were scanned in a range of 25 °C to 200 °C with a heating rate of 10 °C/min, with a constant flow of nitrogen of 50 mL/min. The absorption spectra of the samples were obtained by the Fourier Transform Infrared spectrometer (Perkin Elmer, Billerica, MA, USA) equipped with attenuated total reflectance (ATR). Measurements ranging from 380 to 4000 cm^−1^ at room temperature were established. The automatic signals were collected from 3620 scans at a resolution of 4 cm^−1^ and digitized with the spectrum 10 software.

### 3.14. Statistical Analysis

A completely random design was established. All tests were performed in triplicate. When significant differences (*p* < 0.05) were found between the treatments, Tukey’s test was used to compare the means with a significance of *p* < 0.05.

## 4. Conclusions

The addition of nanoemulsions to gelatine films provided antioxidant activity by incorporating xoconostle and orange oil bioactive compounds. It also modified the structure of the film by improving flexibility due to the interactions of nanoemulsions with structural proteins. These modifications were reflected in the mechanical property measurements (tensile strength, Young’s modulus, and percentage of elongation). Incorporation of nanoparticles into biopolymeric films, such as gelatine films, could improve stability, preservation, and shelf-life, as well as mechanical properties used to produce packaging.

## Figures and Tables

**Table 1:** Antioxidant compounds in gelatine films with different nanoemulsion additions.

Compounds	Films					
	Control	1:0.10	1:0.25	1:0.50	1:0.75	1:1
**Phenols (mg GAE/100 g)**	0.89 ± 0.05 ^a^	10.38 ± 0.92 ^b^	20.86 ± 4.01 ^c^	27.14 ± 3.70 ^d^	34.14 ± 7.08 ^e^	41.32 ± 3.71 ^f^
**Flavonoids (mg QE/100g)**	1.76 ± 0.80 ^a^	8.15 ± 2.20 ^b^	16.57 ± 0.32 ^c^	20.49 ± 2.19 ^d^	23.48 ± 1.11 ^e^	28.03 ± 3.25 ^f^
**% inhibition of ABTS radical**	18.44 ± 9.68 ^a^	62.35 ± 0.24 ^b^	67.23 ± 0.25 ^c^	68.79 ± 0.15 ^c^	71.83 ± 0.12 ^d^	72.13 ± 0.23 ^d^
**% inhibition of DPPH radical**	12.08 ± 0.12 ^a^	63.24 ± 1.90 ^b^	66.92 ± 4.10 ^b^	71.50 ± 1.93 ^b,c^	72.25 ± 5.12 ^b,c^	82.23 ± 2.71 ^d^

Values with the different letter ^a,b,c,d,e,f^ within the rows are statistically different values according to the Tukey test (*p* < 0.05). mg GAE/100 g (mg of gallic acid equivalent per 100 g film); mg QE/100 g (mg of quercetin equivalent per 100 g film); ABTS: inhibition of radical 2,20-azino-bis (3-ethylbenzothiazoline-6-sulphonic acid); DPPH: inhibition of the radical 1,1-diphenyl-2-picrylhydrazyl. All data are expressed as the average value ± standard deviation.

**Table 2:** Effects of xoconostle–orange oil nanoemulsion (W/O) on the color parameters of gelatine films. Brightness: L* value; b* and a*: coordinates.

Treatment	L*	a*	b*
Control	49.66 ± 2.19 ^b^	−0.88 ± 0.05 ^d^	0.71 ± 0.13 ^e^
1:0.1	46.40 ± 2.41 ^a,b^	−1.47 ± 0.09 ^c^	8.16 ± 0.40 ^d^
1:0.25	45.26 ± 0.29 ^a^	−1.49 ± 0.08 ^b^	9.14 ± 0.16 ^d^
1:0.50	45.69 ± 0.51 ^a^	−1.93 ± 0.13 ^a,b^	13.72 ± 0.43 ^c^
1:0.75	45.06 ± 0.63 ^a^	−1.61 ± 0.49 ^b^	15.21 ± 0.31 ^b^
1:1	44.67 ± 1.14 ^a^	−1.23 ± 0.54 ^a^	16.14 ± 0.49 ^a^

Values with the different letter ^a,b,c,d^ in the column are statistically different values according to the Tukey test (*p* < 0.05).
